# Ginsenoside Rg1 Inhibits High Glucose-Induced Proliferation, Migration, and Angiogenesis in Retinal Endothelial Cells by Regulating the lncRNA SNHG7/miR-2116-5p/SIRT3 Axis

**DOI:** 10.1155/2022/6184631

**Published:** 2022-12-03

**Authors:** Liping Xue, Min Hu, Juanjuan Li, Yadi Li, Qin Zhu, Guanglong Zhou, Xiaofan Zhang, Yuan Zhou, Jieying Zhang, Peng Ding

**Affiliations:** ^1^Department of Pediatric Ophthalmology, The Affiliated Hospital of Yunnan University, The Second People's Hospital of Yunnan, The Affiliated Ophthalmology Hospital of Yunnan University, Kunming 650021, Yunnan, China; ^2^Department of Ophthalmology, The Affiliated Hospital of Yunnan University, The Second People's Hospital of Yunnan, The Affiliated Ophthalmology Hospital of Yunnan University, Kunming 650021, Yunnan, China; ^3^Department of Neurosurgery, The First Affiliated Hospital of Kunming Medical University, Kunming 650032, Yunnan, China

## Abstract

**Background:**

Diabetic retinopathy (DR), including retinal angiogenesis and endothelial cell proliferation and migration, is a serious complication in diabetic patients. It has been reported that ginsenoside Rg1 can prevent retinal damage. However, the mechanism by which Rg1 prevents retinal damage is unknown. Therefore, the aim of the present study was to investigate the mechanism by which Rg1 inhibits high glucose-induced complications through the regulation of the lncRNA SNHG7/miR-2116-5p/SIRT3 axis.

**Methods:**

Under high glucose (HG) conditions, human retinal endothelial cells (HRECs) were cultured to simulate a DR environment, and Rg1 was added after 48 h. Negative control (NC), miR-2116-5p mimic, si-SNHG7, pc-DNA SIRT3, and miR-2116-5p inhibitor were transfected into HRECs, and CCK-8 assay was used to detect the cell viability. Angiogenesis and transwell assays were used to evaluate angiogenesis and cell migration, respectively. qRT–PCR and Western blot were used to detect the expression of related genes and proteins. Luciferase reporter assays and bioinformatics were used to analyze the target binding sites of miR-2116-5p to lncRNA SNHG7 and SIRT3.

**Results:**

The proliferation, migration and angiogenesis of HRECs were induced by HG. As expected, HG upregulated miR-2116-5p and VEGF expression but downregulated lncRNA SNHG7 and SIRT3 expression. Importantly, Rg1 inhibited HG-induced HREC proliferation, migration, and angiogenesis by upregulating the lncRNA SNHG7, and miR-2116-5p had a target regulatory relationship with both lncRNA SNHG7 and SIRT3.

**Conclusion:**

Rg1 inhibits HG-induced proliferation, migration, angiogenesis, and VEGF expression in retinal endothelial cells through the lncRNA SNG7/miR-2116-5p/SIRT3 axis. This finding provides theoretical evidence for the clinical application of Rg1 in DR.

## 1. Introduction

Numerous studies have shown that diabetes causes various complications (diabetic nephropathy, diabetic retinopathy (DR) and cardiovascular disease), which have become the main cause of morbidity and mortality of diabetes [1]. Type 2 diabetes can lead to serious neurovascular complications, leading to visual impairment and blindness, and DR is one of the main causes [2]. The basic pathological changes in DR include the selective loss of pericytes, capillary basement membrane thickening, microangioma formation, endothelial cell proliferation, and retinal detachment due to neovascularization [3]. The first barrier to monitoring blood glucose changes is the retinal endothelium. The existing evidence suggests that a high concentration of glucose can lead to increasing numbers and migration of retinal endothelial cells, which is a key step in the occurrence of DR [4]. Although some new drugs and vitreoretinal microsurgery have been used in clinical DR treatment, the incidence of DR has dramatically increased in recent decades [5, 6]. Therefore, further revealing the etiology of DR is important for improving the available treatment methods [7, 8]. Ginsenoside Rg1 (Rg1) is a component of ginsenoside, which mainly exists in ginseng medicinal materials. Rg1 can quickly relieve fatigue, delay aging, stimulate the central nervous system, inhibit platelet aggregation, and improve learning and memory [9]. Rg1 has also been shown to be useful in the treatment of myocardial infarction [10], diabetic limb infarction [11], and ischemic necrosis of the skin [12]. Rg1 promotes neovascularization after myocardial infarction, diabetic limb infarction, skin ischemic necrosis, and neonatal hypoxic encephalopathy [13]. Experimental studies have shown that Rg1 has strong antioxidant and blood glucose-lowering activities [14, 15]. Rg1 can promote angiogenesis and enhance endothelial progenitor cell angiogenesis. Moreover, Rg1 can improve endoplasmic reticulum stress-induced apoptosis in diabetic cardiomyopathy induced by streptozotocin (STZ) [16]. Rg1 prevents retinal damage by inhibiting retinal cell apoptosis [17]. Moreover, experimental studies have shown that ginsenoside Rg1 plays a role in promoting vascular regeneration and enhancing endothelial progenitor cell angiogenesis [18].

LncRNAs have been shown to affect the progression mechanisms of DR through various methods [19]. In human retinal endothelial cells, HG-induced angiogenesis can be inhibited by the lncRNA SNHG7 through the miR-543/SIRT1 cascade [20]. Whether SNHG7 participates in the regulation of vascular growth in DR and whether it promotes angiogenesis in human retinal endothelial cells (HRECs) remain unclear. Notably, in the pathogenesis of DR, miRNAs also play an indispensable role. There have been reports of the abnormal expression of miRNAs in the retina of diabetic rats induced by STZ [21, 22]. In addition, it has been shown that miR-3197 and miR-2116-5p are immensely upregulated in DR patients and are effective diagnostic markers of DR [23].

As a conservative nicotinic adenine dinucleotide-dependent (NAD-dependent) deacetylase, sirtuins consist of seven isomers [24]. In addition, sirtuin-3 (SIRT3), a core member of the sirtuin family, is located on the mitochondrial membrane. SIRT3 can deacetylate the target protein, which plays an important role in antioxidation, biosynthesis, and energy metabolism of mitochondria [25]. For example, under the mediation of SIRT3, autophagy-related proteins can be acetylated, thus affecting autophagy [26]. SIRT3 is necessary for coronary angiogenesis and glycolysis [27]. In type 2 diabetic mice, retinal dysfunction may be related to the loss of SIRT3 and SIRT5 [28] because SIRT3 may promote autophagy by downregulating the expression of angiogenesis-related genes in retinal endothelial cells [29]. In addition, in a rat model of diabetes and retinopathy, the expression of autophagy-related proteins was promoted by the overexpression of SIRT3, while VEGF was inhibited [30]. These findings suggest that SIRT3 is a key therapeutic target for DR.

In the present study, StarBase website prediction suggested that miR-2116-5p has target binding sites for both the lncRNA SNHG7 and SIRT3, implying that miR-2116-5p, lncRNA SNHG7, and SIRT3 may act as an axis. Therefore, we investigated the mechanism by which ginsenoside Rg1 inhibits retinal endothelial cell lesions induced by high glucose by regulating the lncRNA SNHG7/miR-2116-5p/SIRT3 axis.

## 2. Methods

### 2.1. Animal Breeding and Modeling

In total, 120 healthy male SD rats of SPF class (Animal Experiment Center of Kunming Medical University), weighing 200 ± 25 g, were utilized, and all rats had no pathological changes in the anterior and anterior segments of the eyes after the examination. The blood glucose levels were within the normal range as detected by a blood glucose meter after tail vein collection. The rats were randomly divided into 3 groups (40/group): normal rats group (NC); DR rats (Model); Rg1- treated DR rats group (Rg1). The rats were acclimatized and housed for 1 week before the experiment, and they were fed and watered ad libitum. The rats in the diabetic group were fasted for 12 h before modeling and weighed before the experiment. In the diabetic group, freshly prepared STZ in buffer (55 mg/kg; Sigma–Aldrich, Germany) was injected once into the left lower abdominal cavity, and the rats ate and drank normally after the injection. After the rats were injected with STZ for 48 h, the blood glucose and body weight were measured. Additionally, in the diabetic rat models, blood glucose >16.7 mmol/L, polyuria, and polyphagia were considered. The blood glucose and body weight of the rats were observed once every 2 weeks. In the Rg1 group, gavage was started on the day of modeling, and 0.5 mL (12–5 g/ml) of Rg1 solution (Solarbio, Beijing, China) was given by gavage every day, while the same dose of saline was given to the model and NC groups. The rats were sacrificed 8 weeks after modeling, and fresh retinal specimens were removed and preserved for the relevant assays. The experimental scheme of this study was approved by the Animal Ethics Committee of Kunming Medical University and fully met the requirements of the National Institutes of Health Laboratory Animal Care Guide.

### 2.2. Cell Culture and Transfection

HRECs (American Type Culture Collection, Inc.) were cultured at 37°C with saturated humidity for two days before passaging. Before adding 1 mL of trypsin, the cells were washed with PBS 3 times, which covered the entire cell layer. The cells were observed under an inverted microscope until they shrank into a round shape, and then, 10% fetal bovine serum was added to neutralize trypsin. The samples were centrifuged at 1000 rpm for 5 min to collect the cells. Ten percent fetal bovine serum was added, and the cells were inoculated in 75 cm^2^ culture flasks at 10 mL/bottle (10^4^ cells/ml). A concentration of 5 mM glucose is a normal glucose condition, and 25 mM is a high glucose condition. The cells were incubated with 25 mM glucose for 48 h before adding 10 *μ*M Rg1, followed by incubation for 48 h for the subsequent experiments. Using Lipofectamine™ 2000 (Med Chem Express, USA), the negative control (NC), si-SNHG7, miR-2116-5p mimic, miR-2116-5p inhibitor, and pc-DNA SIRT3 were transfected into HRECs.

### 2.3. HE Staining of Retinal Tissue

For the HE staining, rat retinal sections were routinely dewaxed and washed with ddH_2_O for 10 s. Subsequently, hematoxylin staining solution was used to treat the rat retinal sections for 10 min, and the sections were washed with ddH_2_O, subjected to 1% hydrochloric acid alcohol fractionation for 10 s, washed with ddH_2_O, returned to blue in warm water for 1 min, subjected to eosin staining solution for 30 s, washed with water for 10 s, subjected to gradient alcohol dehydration, cleared with xylene, sealed with neutral gum and observed for the detection of inflammatory cell infiltration under a microscope.

### 2.4. Immunohistochemistry

After the glass slide was baked at 65°C for 2 h, it was placed in xylene for 10 minutes. The rat retinal sections were incubated in the following ethanol gradient (5 min per solution): 100%, 95%, 80% and distilled water. In a wet room, citric acid buffer was used to treat the slices, and hydrogen peroxide (3%) was used to remove endogenous peroxidase (25°C, 10 min). The sections were blocked with 5% bovine serum at 37°C for 30 min and then incubated with an anti-SIRT3 antibody (1 : 200) for 12 h at 4°C. The sections were incubated with goat anti-rabbit (IgG, 1 : 100) for 30 min at 37°C after washing the slices with PBS buffer. 3,3′-diaminobenzidine (DAB) was used to observe the sections, and a light microscope was used to acquire the images.

### 2.5. Cell Viability Assay

In total, 5 × 10^4^ cells/well were inoculated into a 96-well plate, 10 *μ*L CCK-8 solution (Sangon, Shanghai, China) was added, and the cells were incubated for 4 h at 25°C. The OD value was measured at 450 nm.

### 2.6. Angiogenesis Experiments

Cells were inoculated into a 24-well plate at 37°C, and Matrigel (Sigma–Aldrich, Germany) was added to each well and allowed to harden for 30 min. HRECs were inoculated at a density of 1.2 × 10^5^ cells/well on top of the Matrigel-coated wells and cultured in a sterile incubator at 100% humidity, 37°C and 5% CO_2_ for 6 h. An inverted microscope was used to observe the tube lumen and acquire the images. Image-Pro Plus software was used to calculate the number of Matrigel tubule formations in the field of view and the tube formation capacity.

### 2.7. Transwell Experiment

Cells were collected from each group 48 h after transfection and washed, and serum-free DMEM was used to adjust the cell concentration to 1 × 10^5^ cells/mL. In 24-well Transwell plates (Corning, USA), 200 *μ*L of cell suspension was added to the upper chamber, and in the lower chamber, 500 *μ*L of DMEM containing 10% fetal bovine serum was added. After culturing for 24 h, the unstained cells were wiped off, while the stained cells were stained with crystal violet for 20 min. An inverted microscope was used to observe the cells, and five randomly selected visual fields were imaged and counted.

### 2.8. qRT-PCR Experiments

The total RNA was extracted from tissues and cells using a Total RNA Extractor (Sangon Biotech). A cDNA synthesis kit (Vazyme, Nanjing, China) was used to reverse transcribe 2 *μ*g mRNA into cDNA, which was then diluted 10 times. One microliter of the prepared cDNA was used for qPCR, and the U6 or GAPDH gene was used as the reference gene. All primers (Table 1) used in this study were designed with Premier 5.0. The two-step reaction conditions for PCR were as follows: predenaturation (maintained at 95°C for 5 min), maintenance at 95°C for 10 s, annealing (30 s) and extension (30 s). Both annealing and extension were cycled 40 times. The confidence of the PCR results was assessed by the dissociation curve and cycle threshold (CT) values. The results were calculated by the 2^−ΔΔCt^ method after repetition at least 3 times.

### 2.9. Western Blot Assay

Proteins were extracted from retinal tissue utilizing RIPA lysis buffer (Sangon Biotech, Shanghai), and a BCA assay (Sangon Biotech, Shanghai) was used to determine the total protein content. 10% SDS‒PAGE gel was used to separate the total proteins, which were then transferred to PVDF membranes by a constant current flow at 200 mA. Subsequently, the PVDF membranes were incubated with antibodies (Abcam, USA) for 12 h at 4°C. The PVDF membranes were washed with TBS buffer and incubated with secondary antibodies at 25°C for 1 h. After washing the membranes three times, chemiluminescent reagents were added, and the bands were analyzed for grayscale values using ImageJ software. Each experiment was repeated 3 times independently.

### 2.10. Bioinformatics and Dual Luciferase Gene Reporter Analysis

In this study, StarBase (http://starbase.sysu.edu.cn/) was used to predict the binding sites of miRNAs and lncRNAs. The dual-luciferase reporter vectors containing WT and mutant-type binding sites for SNHG7 or SIRT3 sequences were constructed by a rapid cloning kit (Vazyme, Nanjing, China) and named WT-SNHG7 or WT-SIRT3 and MUT- SNHG7 or SIRT3, respectively. Subsequently, WT-SNHG7 or WT-SIRT3 and MUT-SNHG7 or SIRT3 vectors were transfected into HRECs (Chinese Academy of Sciences Culture Collection) with NC mimic or miR-29b-3p mimic. After transfection for 48 h, a dual luciferase reporter assay was used to detect luciferase activity.

### 2.11. Statistical Analysis

GraphPad Prism 8 software was used to analyze and prepare graphs of the experimental data. In this study, the results are shown as the mean ± standard deviation (SD). As expected, two groups and multiple groups of data were analyzed by unpaired Student's *t*-test and one-way analysis of variance, followed by Tukey's post-hoc test. The *P* value representing statistical significance was 0.05.

## 3. Results

### 3.1. Effect of Rg1 on the Expression of SIRT3, the lncRNA SNHG7, and miR-2116-5p in the Retina of DR Rats

**Figure 1:**
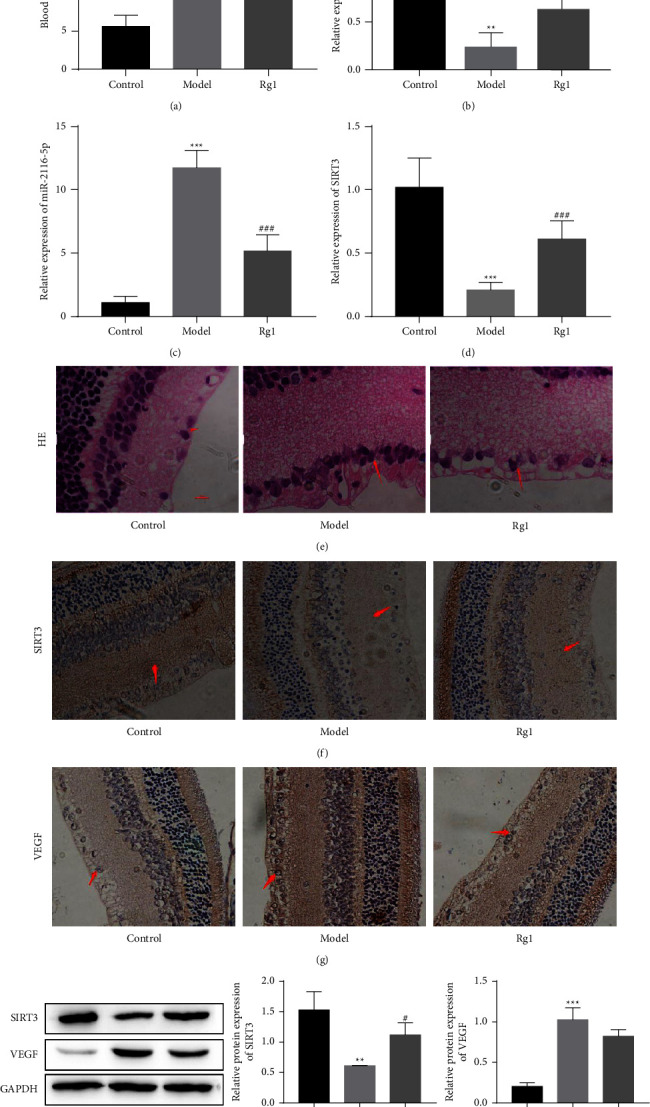
The effect of Rg1 on the lncRNA SNHG7, miR-2116-5p, and SIRT3 in the retinas of DR rats. (a) Blood glucose values of rats after different treatments. qRT-PCR was used to analyze the expression of the lncRNA SNHG7 (b), miR-2116-5p (c), and SIRT3 (d). (e) HE staining of rat retinal tissues. Immunohistochemical staining of SIRT3 (f) and VEGF (g). (h) SIRT3 and VEGF protein levels were detected by Western blot analysis. ^*∗∗*^*P* < 0.01 and ^*∗∗∗*^*P* < 0.001 compared to the control group; ^#^*P* < 0.05, ^##^*P* < 0.01 and ^###^*P* < 0.001 compared to the model group.

The effect of Rg1 on the expression of SIRT3, the lncRNA SNHG7 and miR-2116-5p in the retina of DR rats was investigated. Compared with the control group, the detection of blood glucose values in the different treatment groups revealed that the model group rats had significantly higher blood glucose after the STZ injection, and the blood glucose level was higher than 16.7 mmol/L, demonstrating a successful diabetic model. In contrast, the treatment group had significantly lower blood glucose (Figure 1(a)). Compared with the control group, SNHG7 and SIRT3 were significantly lower in the model group, and the expression of both SNHG7 and SIRT3 increased after the Rg1 treatment as shown by qRT-PCR (Figures 1(b) and 1(d)). As expected, compared with the control group, the expression of miR-2116-5p was significantly higher in the model group, and the expression of miR-2116-5p was significantly lower after the Rg1 treatment (Figure 1(c)). The HE staining results showed that the control rats had a clear and continuous inner boundary membrane and only a few vascular endothelial cells in the vitreous near the inner retinal boundary membrane. The model rats showed edema on the retinal surface, and the number of vascular endothelial cells was considerably increased. Moreover, the rats in the Rg1-treated group had a clear and continuous inner boundary membrane, reduced edema and decreased vascular endothelial cells (Figure 1(e)). Compared to the control group, SIRT3 was significantly reduced in the retinal tissues of the rats in the model group in the immunohistochemical assay. In contrast, SIRT3 in the Rg1-treated rats was significantly higher than that in the model group (Figure 1(f)). The VEGF-immunopositive product was indicated by brownish-yellow granular staining, and immunopositive cells were mainly distributed in the retinal ganglion cell layer, which was opposite to that observed with SIRT3 (Figure 1(g)), and in the inner nuclear layer. The results of Western blot detection also showed that compared with the control group, the expression of SIRT3 was down-regulated and VEGF was up-regulated in the model group, and Rg1 treatment reversed this phenomenon (Figure 1(h)). In summary, these findings show that Rg1 downregulates miR-2116-5p and VEGF but upregulates the lncRNA SNHG7 and SIRT3 in the retinas of diabetic rats.

### 3.2. Effect of Rg1 on the Proliferation, Migration, and Angiogenesis of HG-Treated HRECs

**Figure 2:**
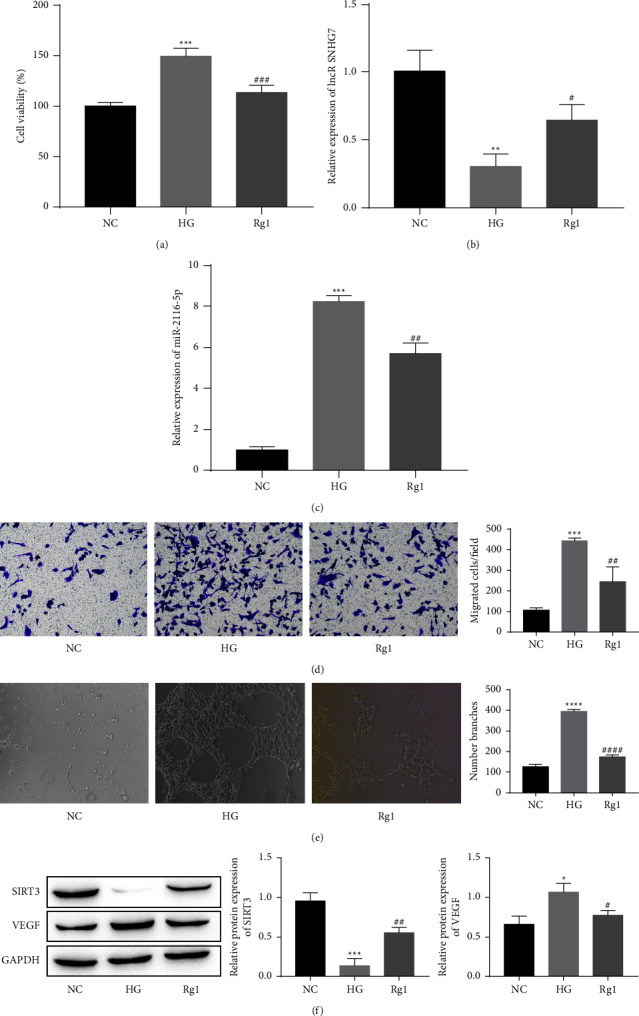
Effect of Rg1 on the proliferation, migration, and angiogenesis of HRECs under HG-induced conditions. (a) CCK-8 assay of cell viability. Qrt-PCR analysis of the lncRNA SNHG7 (b) and miR-2116-5p (c). (d) Transwell assay of cell migration. (e) Comparison of angiogenesis in different groups. (f) Analysis of the SIRT3 and VEGF protein levels by a Western blot analysis. ^*∗*^*P* < 0.05, ^*∗∗*^*P* < 0.01, ^*∗∗∗*^*P* < 0.001 and ^*∗∗∗∗*^*P* < 0.0001 compared to the NC group; ^#^*P* < 0.05, ^##^*P* < 0.01, ^###^*P* < 0.001 and ^####^*P* < 0.0001 compared to the HG group.

Cell viability was assessed using a CCK-8 assay to investigate the effect of Rg1 on HG-induced pathological phenomena (HREC proliferation, migration and angiogenesis). The results showed that the HG treatment significantly increased the viability of HRECs, while the Rg1 treatment significantly inhibited cell viability (Figure 2(a)). As expected, in the HG group, the qRT–PCR analysis showed that the lncRNA SNHG7 and miR-2116-5p were significantly lower and higher, respectively, and they were significantly reversed after the addition of Rg1 (Figures 2(b) and 2(c)). The number of migrating cells and angiogenesis were significantly higher in the HG group in the Transwell and angiogenesis assays, and the number of migrating cells and angiogenesis were decreased after the addition of Rg1 (Figures 2(d) and 2(e)). Similarly, the protein expression of SIRT3 was significantly lower and VEGF was elevated in the HG group as shown by the Western blot analysis. The treatment with Rg1 significantly increased the protein level of SIRT3 but significantly decreased VEGF (Figure 2(f)). Thus, these findings demonstrate that high glucose induces pathological phenomena in HRECs, but Rg1 significantly inhibits these changes.

### 3.3. The Targeting Relationship between the lncRNA SNHG7 and miR-2116-5p

**Figure 3:**
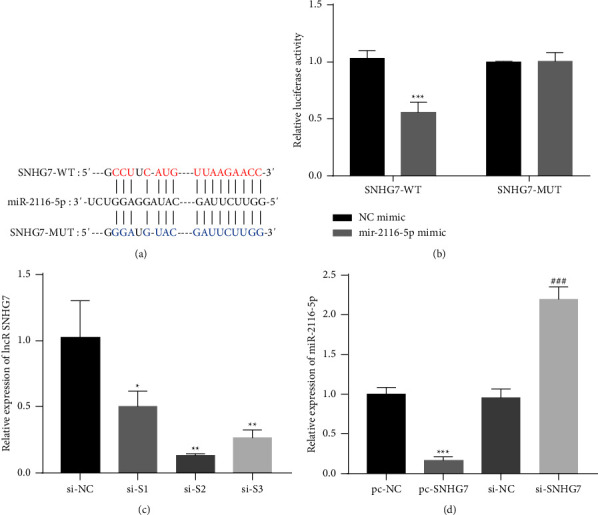
Validation of the relationship between the lncRNA SNHG7 and miR-2116-5p. (a) The predicted lncRNA SNHG7-binding sites in miR-2116-5p. (b) The targeting relationship between the lncRNA SNHG7 and miR-2116-5p was verified through a dual luciferase reporter assay, ^*∗∗∗*^*P* < 0.001 compared to the NC mimic group. (c) Analysis of the lncRNA transfection efficiency of SNHG7 by qRT-PCR, ^*∗*^*P* < 0.05 and ^*∗∗*^*P* < 0.01 compared to the si-NC group. (d) The expression of miR-2116-5p was detected by qRT–PCR, ^*∗∗∗*^*P* < 0.001 compared to the pc-NC group; ^###^*P* < 0.001 compared to the si-NC group.

StarBase online software was used to predict the binding sites of lncRNA SNHG7 in miR-2116-5p (Figure 3(a)). As verified by the dual luciferase assays, the luciferase activity of wild-type SNHG7 could be reduced by miR-2116-5p but had almost no effect on mutant SNHG7 (Figure 3(b)). The transfection of different siRNAs, including siRNA NC (si-NC) and siRNA-SNHG7 (si-S1/2/3), was used to knockdown SNHG7. Because the transfection of si-S2 showed the best knockdown of SNHG7, it was used in the subsequent experiments (Figure 3(c)). The knockdown or overexpression of SNHG7 was verified by a qRT-PCR analysis. The results showed that miR-2116-5p was significantly decreased after the overexpression of SNHG7, while miR-2116-5p was significantly increased after the knockdown of SNHG7 (Figure 3(d)). Thus, these findings demonstrate that the lncRNA SNHG7 negatively regulates miR-2116-5p by targeting the modulation of miR-2116-5p.

### 3.4. Rg1 Inhibits HG-Induced Cell Proliferation, Migration, and Angiogenesis by Upregulating the lncRNA SNHG7 in HRECs

**Figure 4:**
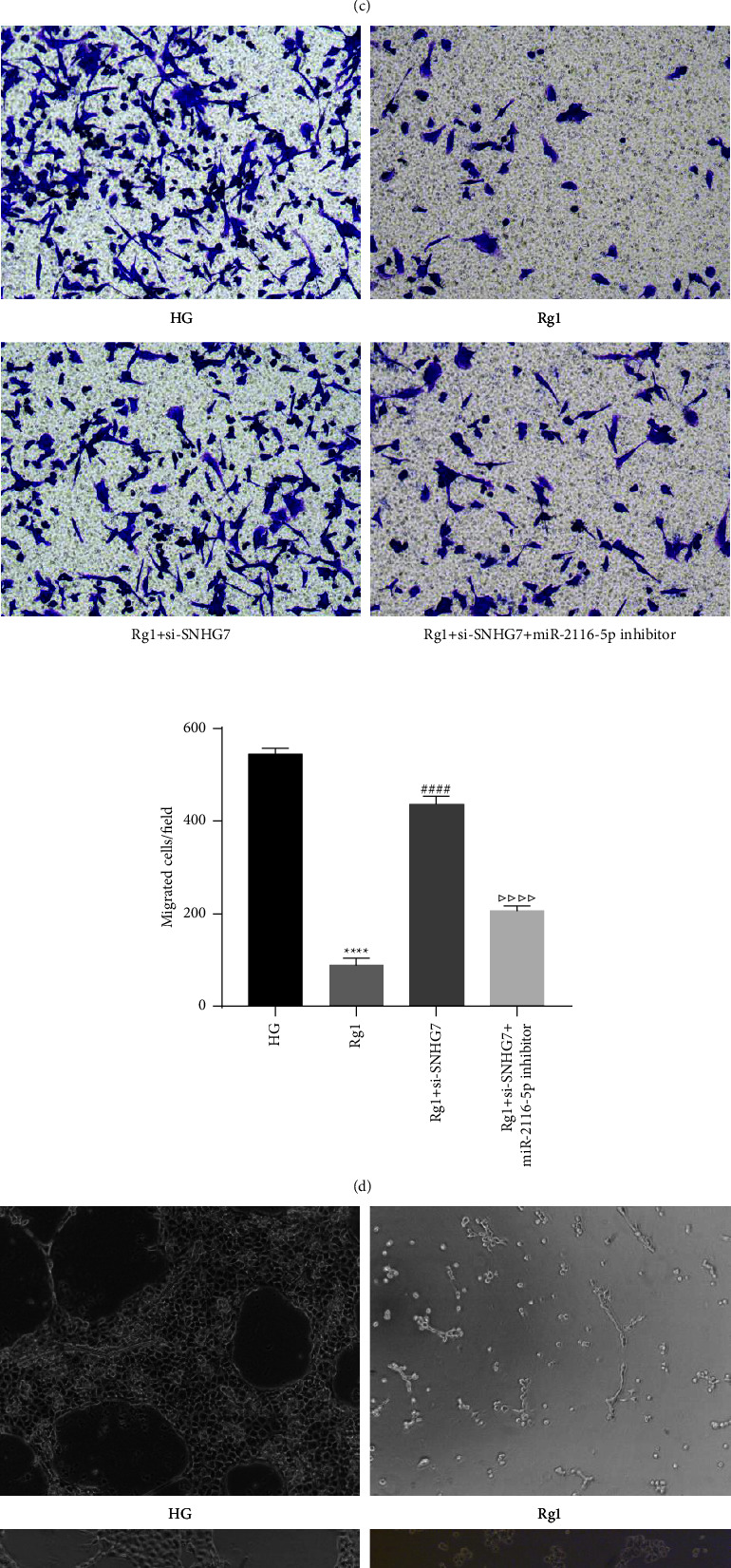
Rg1 inhibits HG-induced cell proliferation, migration, and angiogenesis in HRECs through the upregulation of the lncRNA SNHG7. (a) CCK-8 assay of cell viability. qRT-PCR analysis of the expression level of the lncRNA SNHG7 (b) and miR-2116-5p (c). (d) Transwell assay of cell migration. (e) Comparison of angiogenesis in different groups. ^*∗∗∗∗*^*P* < 0.0001 compared to the HG group; ^####^*P* < 0.0001 compared to the Rg1 group; ^△^*P* < 0.05, ^△△^*P* < 0.01 and ^△△△△^*P* < 0.0001 compared to- the Rg1+si-SNHG7 group.

Next, we investigated the effects of Rg1 in HG-induced HRECs via the lncRNA SNHG7. Compared with the HG group, the cell viability was reduced in the Rg1 group, however, si-SNHG7 reversed the inhibitory effect of Rg1 on cell proliferation. Furthermore, compared with the Rg1+si-SNHG7 group, the cell viability was significantly reduced in the Rg1+si-SNHG7+miR-2116-5p inhibitor group (Figure 4(a)). The results of qRT-PCR assay showed that compared with the HG group, the expression of SNHG7 was significantly increased and the expression of miR-2116-5p was significantly down-regulated in the Rg1 group, which was reversed by si-SNHG7. At the same time, compared with the Rg1+si-SNHG7 group, in the Rg1+si-SNHG7+miR-2116-5p inhibitor group, the expression of SNHG7 was up-regulated and the expression of miR-2116-5p was down-regulated (Figures 4(b) and 4(c)). Transwell and angiogenesis experiments showed that Rg1 treatment could effectively inhibit HG-induced cell proliferation and angiogenesis, while knockdown of SNHG7 could significantly attenuate the effect of Rg1. In addition, co-transfection of si-SNHG7+miR-2116-5p inhibitor could maintain the inhibitory effect of Rg1 on cell proliferation and angiogenesis to a certain extent (Figures 4(d) and 4(e)). These results suggest that Rg1 inhibits HG-induced HREC pathological phenomena through the upregulation of the lncRNA SNHG7.

### 3.5. Validation of the Targeting Relationship between miR-2116-5p and SIRT3

**Figure 5:**
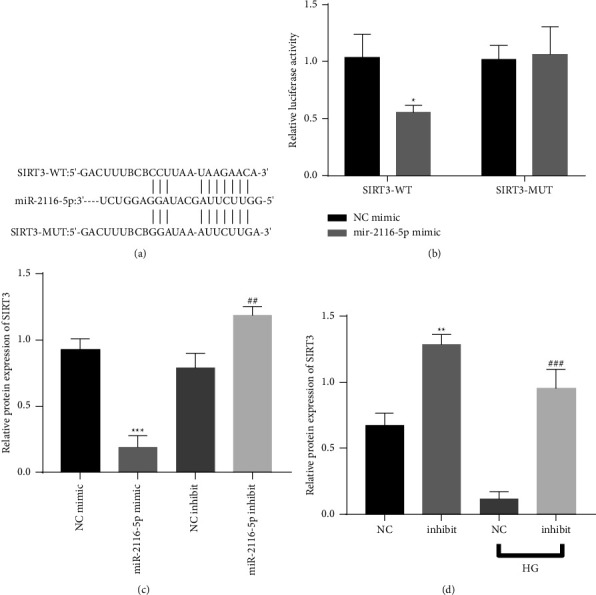
Validation of the relationship between miR-2116-5p and SIRT3. (a): The predicted miR-2116-5p binding sites in SIRT3. (b): Verification of the relationship between miR-2116-5p and SIRT3 by a dual luciferase reporter assay, ^*∗*^*P* < 0.05 compared to the NC mimic group. (c) Analysis of the transfection efficiency of SIRT3 by qRT-PCR, ^*∗∗∗*^*P* < 0.001 compared to the NC mimic group; ^##^*P* < 0.01 compared to the NC inhibitor group. (d) The expression of SIRT3 was detected by qRT–PCR. ^*∗∗*^*P* < 0.01 compared to the NC group; ^###^*P* < 0.001 compared to the NC-HG group.

StarBase online software was used to predict the miR-2116-5p-binding sites in SIRT3, and the results are shown in Figure 5(a). As verified by the dual luciferase assays, miR-2116-5p reduced the activity of wild-type SIRT3 but had almost no effect on mutant SIRT3 (Figure 5(b)). SIRT3 was decreased after the transfection of the miR-2116-5p mimic, and the transfection of the miR-2116-5p inhibitor increased the SIRT3 expression levels (Figure 5(c)). At the expression level, SIRT3 was reduced under high glucose conditions and after the transfection of miR-2116-5p under normal glucose and HG conditions (Figure 5(d)). Thus, these data illustrate that miR-2116-5p acts by targeting the negative regulation of SIRT3.

### 3.6. Rg1 Affects the Proliferation, Migration and Angiogenesis of HG-Induced HRECs via miR-2116-5p/SIRT3

**Figure 6:**
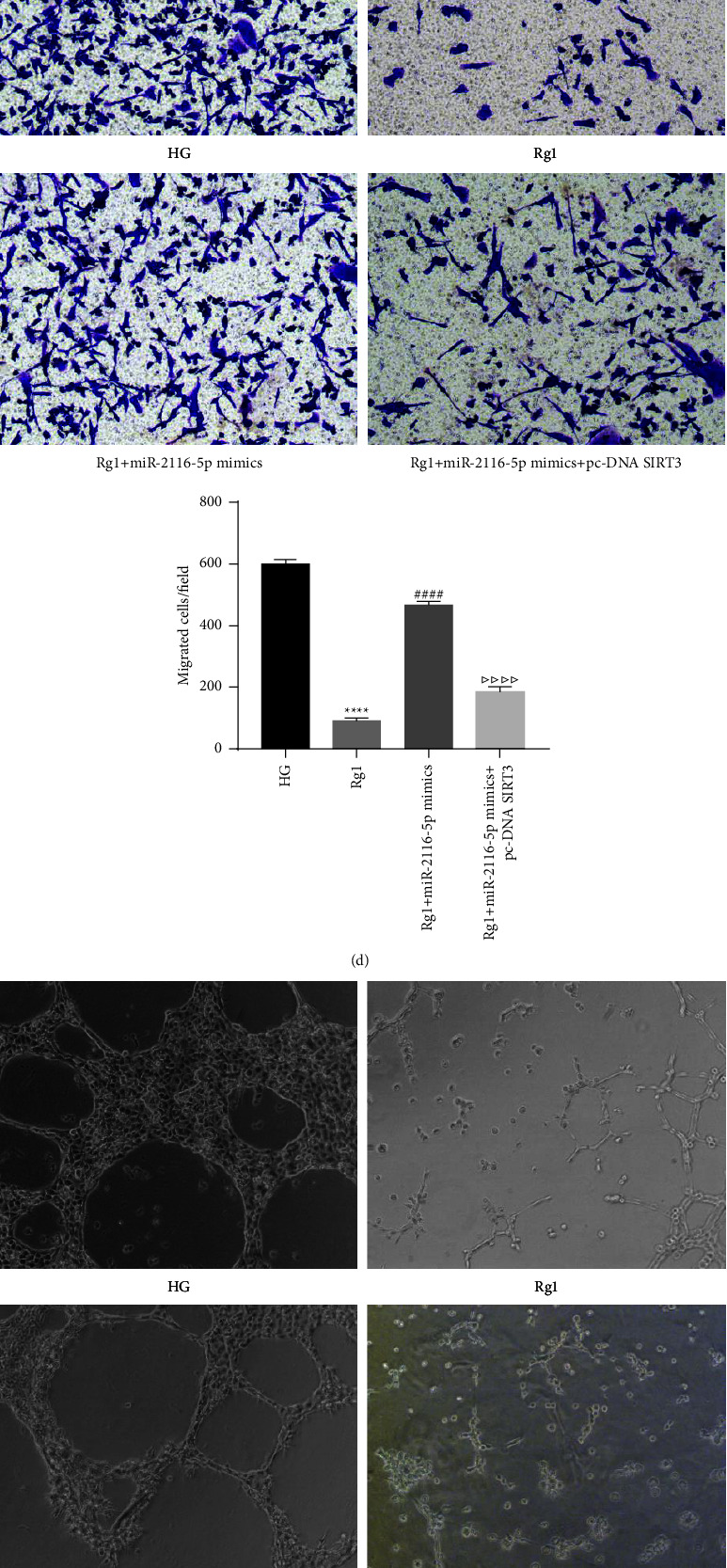
Rg1 affects the proliferation, migration and angiogenesis of HG-induced HRECs through miR-2116-5p/SIRT3. (a) CCK-8 assay of cell viability. qRT-PCR analysis of miR-2116-5p (b) and SIRT3 (c) expression. (D) Transwell assay of cell migration. (E) Comparison of angiogenesis in different groups. (f) Analysis of the SIRT3 and VEGF protein levels by a western blot analysis. ^*∗∗∗*^*P* < 0.001 and ^*∗∗∗∗*^*P* < 0.0001 compared to the HG group; ^##^*P* < 0.01 and ^####^*P* < 0.0001 compared Rg1 group; ^△^*P* < 0.05, ^△△^*P* < 0.01 and ^△△△△^*P* < 0.0001 compared to the Rg1+miR-2116-5p mimic group.

We further explored the effects of Rg1 via miR-2116-5p/SIRT3. The results of CCK-8 assay showed that compared with the HG group, the cell viability of the Rg1 group was reduced, but the transfection of miR-2116-5p mimic reversed the inhibitory effect of Rg1 on cell proliferation to a certain extent. In addition, compared with the Rg1 + miR-2116-5p mimic group, the Rg1 + miR-2116-5p mimic + pc-DNA SIRT3 group had lower cell proliferation activity (Figure 6(a)). The results of qRT-PCR assay showed that compared with the HG group, Rg1 could inhibit the expression of miR-2116-5p and promote the expression of SIRT3, but this phenomenon was reversed by transfection of miR-2116-5p mimic. Meanwhile, compared with the Rg1+miR-2116-5p mimic group, the expression of miR-2116-5p was down-regulated and the expression of SIRT3 was up-regulated in the Rg1+miR-2116-5p mimic + pc-DNA SIRT3 group (Figures 6(b) and 6(c)). The results of Transwell and angiogenesis assays showed that the inhibitory effect of Rg1 on cell proliferation and angiogenesis could be reversed by transfection of miR-2116-5p mimic, but co-transfection of miR-2116-5p mimic + pc-DNA can maintain the inhibitory effect of Rg1 on cell proliferation and angiogenesis to a certain extent (Figures 6(d) and 6(e)). Similarly, Western blot detection results showed that the promoting effect of Rg1 on SIRT3 expression and the inhibitory effect of VEGF expression were reversed by the transfection of miR-2116-5p mimic, but the transfection of miR-2116-5p mimic + PC-DNA SIRT3 maintained this effect of Rg1 to a certain extent (Figure 6(f)). Thus, these findings demonstrate that Rg1 affects the proliferation, migration, and angiogenesis of HG-induced HRECs via miR-2116-5p/SIRT3.

## 4. Discussion

Retinopathy caused by diabetes is a serious ocular complication that mainly manifests as retinal endocrine and hematological damage [31]. Hyperglycemia and hyperlipidemia are direct factors in the development of DR [32]. Endothelial cell damage caused by HG is one of the main clinical features of DR; therefore, endothelial cell activity regulation-related molecules are considered to play a key role in the pathogenesis of DR [33]. In diabetes modeling, higher blood glucose concentrations are an important marker of success [34]. During the construction of the diabetes model in this study, we successfully constructed a diabetic rat model because the blood glucose concentration of the rats was higher than 16.7 mmol/L. One month after the onset of diabetes, peripapillary cell degeneration, retinal thickness, and retinal apoptosis were reduced in the diabetic rats [35]. In this study, the pathological features of the retinal tissue in the rats with diabetes mellitus were described, and new blood vessels were observed in the diabetic retina [36]. Moreover, there was a significant increase in cellular angiogenesis in HRECs under HG induction and a significant increase in cell viability and migration. As a key factor in diabetes, Rg1 can protect molecules from damage. In diabetic rats treated with Rg1, cardiomyocyte apoptosis is inhibited, and caspase 3 expression is downregulated [37]. In the present study, Rg1 had a protective effect on the retina of DR rats and HRECs under HG induction. Regarding the gene expression level, Rg1 increased SIRT3 but decreased VEGF in rat retinal tissue and inhibited HRECs proliferation, migration and angiogenesis. It is consistent with the finding by Gao et al. [17] that Rg1 can prevent DR by reducing apoptosis.

The lncRNA SNHG7 was reduced in HRMECs under HG stimulation, and lncRNA SNHG7 overexpression inhibited HG-induced pathological phenomena (cell migration, proliferation and angiogenesis) by regulating the miR-543/SIRT1 axis [20]. Our study also demonstrated that HG conditions downregulated SNHG7 and its inhibitory effect on HG-induced pathological phenomena. There is a targeted binding site between SNHG7 and miR-2116-5p, and the inhibition of miR-2116-5p can effectively attenuate the effect of knockdown of SNHG7 on the proliferation and angiogenesis of RG1 cells. Furthermore, we found that the target of miR-2116-5p is SIRT3. As expected, as a downstream pathway of SNHG7, miR-2116-5p/SIRT3 mediated its protective effect on HRECs, while Rg1 functioned by upregulating SNHG7 to regulate the miR-2116-5p/SIRT3 axis. As a result, these findings show that SIRT3 may play a role in regulating neovascularization [29]. The overexpression of SIRT3 has been shown to inhibit retinal neovascularization under HG and insulin-induced conditions [29]. Our study found that overexpression of SIRT3 could reverse the promoting effect of miR-2116-5p on angiogenesis, which also indicated that SIRT3 could inhibit angiogenesis. In this study, SIRT3 was significantly reduced after the development of DR. VEGF can maintain ocular vascular integrity, and its expression is low and necessary in normal healthy eyes [38]. However, in DR, the levels of VEGF are higher than normal in cells and body fluids. Elevated VEGF levels alter capillary permeability, leading to retinal neovascularization, retinal vascular hemorrhage, exudation and increased angiogenesis and visual impairment. Importantly, inhibiting the expression of VEGF can inhibit the formation of retinal neovascularization [39]. This study shows that in the DR model, VEGF expression was increased. However, VEGF can be inhibited by SIRT3 overexpression, which may affect the formation of new blood vessels in the retina by regulating VEGF expression to protect against retinal injury.

In summary, the present study investigated the molecular mechanisms related to the alleviation of DR by Rg1. We demonstrated that Rg1 inhibits HG-induced cell proliferation, migration and angiogenesis and VEGF expression in retinal endothelial cells through the lncRNA SNHG7/miR-2116-5p/SIRT3 axis. These findings provide a theoretical basis for the clinical use of Rg1 for the treatment of DR. In addition, our study has the limitation of not verifying our molecular mechanism in vivo experiments. In the next study, we will verify that Rg1 alleviates DR through the lncRNA SNHG7/miR-2116-5p/SIRT3 axis in animal experiments.

## Figures and Tables

**Table 1:** Primer sequences.

Target	Sequence
SNHG7	Forward: 5′-GCCCTGCAGCCTCGC-3′
	Reversed: 5′-CAGCGGCGCCTCCTC-3′

miR-2116-5p	Forward: 5′-GGGTTCTTAGCATAGGAGGTC-3′
	Reversed: 5′-GAATCGAGCACCAGTTACGCAATG-3′

SIRT3	Forward: 5′- CAATGTCGCTCACTACTTCCTT-3′
	Reversed: 5′- CGTCAGCCCGTATGTCTTC-3′

U6	Forward: 5′-CTCGCTTCGGCAGCACA-3′
	Reversed: 5′-AACGCTTCACGAATTTGCGT-3′

GAPDH	Forward: 5′-AATCCCATCACCATCTTCCA-3′
	Reversed: 5′-TGGACTCCACGACGTACTCA-3′

## Data Availability

The datasets used and/or analyzed during the current study are available from the corresponding author upon reasonable request.
